# TGFβ Signaling Increases Net Acid Extrusion, Proliferation and Invasion in Panc-1 Pancreatic Cancer Cells: SMAD4 Dependence and Link to Merlin/NF2 Signaling

**DOI:** 10.3389/fonc.2020.00687

**Published:** 2020-05-07

**Authors:** Raj R. Malinda, Katrine Zeeberg, Patricia C. Sharku, Mette Q. Ludwig, Lotte B. Pedersen, Søren T. Christensen, Stine F. Pedersen

**Affiliations:** Section for Cell Biology and Physiology, Department of Biology, Faculty of Science, University of Copenhagen, Copenhagen, Denmark

**Keywords:** NHE1, SLC9A1, NBCn1, SLC4A7, Merlin, proliferation, invasion, PDAC

## Abstract

Pancreatic ductal adenocarcinoma (PDAC) is a major cause of cancer-related death, with a 5-year survival of <10% and severely limited treatment options. PDAC hallmarks include profound metabolic acid production and aggressive local proliferation and invasiveness. This phenotype is supported by upregulated net acid extrusion and epithelial-to-mesenchymal transition (EMT), the latter typically induced by aberrant transforming growth factor-β (TGFβ) signaling. It is, however, unknown whether TGFβ-induced EMT and upregulation of acid extrusion are causally related. Here, we show that mRNA and protein expression of the net acid extruding transporters Na^+^/H^+^ exchanger 1 (NHE1, SLC9A1) and Na^+^, HCO${}_{3}^{-}$ cotransporter 1 (NBCn1, SLC4A7) are increased in a panel of human PDAC cell lines compared to immortalized human pancreatic ductal epithelial (HPDE) cells. Treatment of Panc-1 cells (which express SMAD4, required for canonical TGFβ signaling) with TGFβ-1 for 48 h elicited classical EMT with down- and upregulation of epithelial and mesenchymal markers, respectively, in a manner inhibited by SMAD4 knockdown. Accordingly, less pronounced EMT was induced in BxPC-3 cells, which do not express SMAD4. TGFβ-1 treatment elicited a SMAD4-dependent increase in NHE1 expression, and a smaller, SMAD4-independent increase in NBCn1 in Panc-1 cells. Consistent with this, TGFβ-1 treatment led to elevated intracellular pH and increased net acid extrusion capacity in Panc-1 cells, but not in BxPC-3 cells, in an NHE1-dependent manner. Proliferation was increased in Panc-1 cells and decreased in BxPC-3 cells, upon TGFβ-1 treatment, and this, as well as EMT *per se*, was unaffected by NHE1- or NBCn1 inhibition. TGFβ-1-induced EMT was associated with a 4-fold increase in Panc-1 cell invasiveness, which further increased ~10-fold upon knockdown of the tumor suppressor Merlin (Neurofibromatosis type 2). Knockdown of NHE1 or NBCn1 abolished Merlin-induced invasiveness, but not that induced by TGFβ-1 alone. In conclusion, NHE1 and NBCn1 expression and NHE-dependent acid extrusion are upregulated during TGFβ-1-induced EMT of Panc-1 cells. NHE1 upregulation is SMAD4-dependent, and SMAD4-deficient BxPC-3 cells show no change in pH_i_ regulation. NHE1 and NBCn1 are not required for EMT *per se* or EMT-associated proliferation changes, but are essential for the potentiation of invasiveness induced by Merlin knockdown.

## Introduction

Pancreatic ductal adenocarcinoma (PDAC) is one of the most devastating cancers globally (1). The exceedingly poor prognosis for PDAC patients reflects a combination of late detection, rapid local invasiveness, and a severe lack of reliable biomarkers and efficacious treatment schemes (2). PDAC is associated with extensive metabolic changes, and PDAC tumors are accordingly highly acidic (3). While PDAC genotypes are highly complex, the most widely characterized driver mutations are activating *KRAS* mutations, inactivating *p53* tumor suppressor mutations, and inactivation or loss of the cyclin-dependent kinase inhibitor 2A (*CDKN2A, P16INK4*) and the transforming growth factor β (TGF-β) effector, *SMAD4* (4, 5).

TGFβ signaling involves the binding of a TGFβ dimer (TGFβ-1,−2, or−3, of which TGFβ-1 is most ubiquitous) to the TGFβ receptor types I and II (TGFβRI and –II; the former also known as ALK5). This results in formation of a hetero-tetrameric receptor complex, where TGFβRII phosphorylates and activates TGFβRI. TGFβRI in turn phosphorylates the transcription factors SMAD2/3, which bind to the co-SMAD, SMAD4, to form a hetero-trimeric protein complex that enters the nucleus to control gene expression. This complex may further interact with a variety of other transcription factors, which are necessary cofactors for SMAD-dependent gene regulation (6, 7). TGFβ ligands also signal through SMAD-independent pathways, including mitogen-activated protein kinases, small GTPases, and the phosphatidyl-inositol-3-kinase (PI3K)-AKT-mTOR pathway (6, 7).

In non-cancer epithelial cells and in premalignant cells, TGFβ signaling is consistently cytostatic, blocking cell cycle progression by increased expression of cyclin-dependent kinase (CDK) inhibitors. However, in many cancer cells, this is overridden by strong CDK activation by other pathways, causing TGFβ to be pro-tumorigenic (6). Accordingly, TGFβ signaling has been shown to stimulate cell motility, invasion, and proliferation, and limit antitumor immune response, and TGFβRI inhibition can revert these effects (8–10). Both pro- and antitumorigenic, highly genotype-dependent roles of TGFβ signaling were demonstrated in PDAC cells (4, 11–13). Illustrating the importance of TGFβ signaling in this cancer, a recent study showed that almost 50% of PDAC patient tumors exhibited mutations in TGF-β signaling components. While *SMAD4* inactivating mutations are most common, mutations in *SMAD3*, TGFβ receptor type I (*TGFBR1)* and−2 *(TFGBR2)* are also reported (4).

TGFβ signaling is a major driver of epithelial-to-mesenchymal transition (EMT), a process with key roles in metastasis and chemotherapy resistance (6, 8, 11, 14–16). In PDAC, TGFβ-induced EMT has been reported to involve SMAD4-dependent (17) and -independent (18) signaling, however, the process is incompletely understood.

Solid tumors are characterized by an often profoundly acidified extracellular pH (pH_e_), a neutral or slightly increased intracellular pH (pH_i_), and a greatly increased rate of acid extrusion (19, 20). The latter occurs because the acid generated by the high, predominantly glycolytic, metabolism of tumor cells is actively extruded from the cancer cells by specific transporters. These transporters, including the Na^+^/H^+^ exchanger NHE1 (SLC9A1) and the Na^+^, HCO${}_{3}^{-}$ cotransporters NBCn1 (SLC4A7) and NBCe2 (SLC4A5) confer additional advantages to the cancer cells, including stimulation of proliferation, survival, and invasiveness, leading to increased tumor growth and metastasis (21–24). In particular NHE1 is important for cell motility and invasiveness, which are key downstream events in EMT (25). Directly implying a link to TGFβ, NHE1 is implicated in fibronectin release in a manner rescued by TGFβ-1 (26).

We therefore hypothesized that net acid extruding proteins are regulated by TGFβ signaling in human PDAC cells and contribute to its downstream effects. We here show that TGFβ-1-induced EMT of Panc-1 cells is associated with increased protein levels of NHE1 and NBCn1 as well as increased pH_i_, whereas smaller changes were observed in SMAD4-deficient BxPC-3 cells, which show only a very modest EMT. This difference between the two cell lines is corroborated in the opposite effects of TGFβ-1 on proliferation, which is increased in Panc-1 and decreased in BxPC-3 cells. Furthermore, knockdown of the tumor suppressor Merlin potentiates TGFβ-1-induced Panc-1 cell invasiveness in a manner dependent on both NHE1 and NBCn1. We propose that acid-extruding transporters are novel players in TGFβ-1-induced EMT in PDAC cells.

## Materials and Methods

### Antibodies and Reagents

Primary antibodies used in western blot analysis were: mouse anti-β-actin, mouse anti-α-tubulin, and mouse anti-α-smooth muscle actin (α-SMA), all from Sigma-Aldrich; goat polyclonal anti-CTGF and mouse anti-NHE1 (clone 54), anti-Poly-ADP Ribose Polymerase (PARP), anti-cleaved PARP (Asp214) and anti-pSer807/811-Rb, all from Santa Cruz Biotechnology; mouse anti-dynactin 1 (DCTN1) and mouse anti-E-cadherin, from BD Biosciences; rabbit anti-GAPDH, rabbit anti-Histone 3, rabbit anti-Merlin, mouse anti-p53, mouse anti-Ki67, and rabbit anti-β-catenin, all from Cell Signaling. Mouse anti- Ki-67 was from Dako (Glostrup, Denmark) and rabbit polyclonal anti-NBCn1 was a kind gift from Jeppe Prætorius, Aarhus University, Denmark. Secondary antibodies used in western blotting were horseradish-peroxidase-conjugated goat polyclonal anti-mouse or anti-rabbit and rabbit polyclonal anti-goat from Dako. Secondary antibodies for immunofluorescence analysis were AlexaFluor488-conjugated donkey anti-mouse or anti-rabbit, and AlexaFluor568-conjugted donkey anti-mouse or anti-rabbit, all from Invitrogen. Recombinant human TGFβ-1 (PHG9214) was from Life Technologies. Mouse monoclonal antibody against Proliferating cell nuclear antigen (PCNA) was from Cell Signaling Technology.

### Cell Culture

Human PDAC cell lines MIAPaCa-2, Panc-1, BxPC-3, and AsPC-1 were acquired from American Type Culture Collection (ATCC, Rockville, MD, USA) and maintained in RPMI medium 1640+GlutaMAX^TM^-I or Dulbecco's Modified Eagle's medium (DMEM)+GlutaMAX^TM^-I (both from Gibco) supplemented with 10% (v/v) fetal bovine serum and 100 U/ml penicillin and 100 μg/mL streptomycin at 37°C in a humidified atmosphere of 5% CO_2_. MIAPaCa-2 cell medium was further supplemented with 2.5% (v/v) heat-inactivated horse serum. Immortalized human pancreatic ductal epithelial *(*HPDE H6c7*)* cells were a kind gift from Dr. Ming-Sound Tsao at Ontario Cancer Institute, Toronto, Canada (27, 28) and were cultured in kerantinocyte basal medium supplemented with epidermal growth factor and bovine pituitary extract.

### siRNA-Mediated Knockdown

siRNAs employed were: NHE1 siRNA: ON-TARGET SMART pool (Thermo Scientific); NBCn1 siRNA (SASI_Hs01_00030755, Sigma-Aldrich) sense sequence 5′-CAUUAACUGGGAUUGCCUA-3′, Merlin siRNA (SASI_Hs_01_00188862, Sigma) sense sequence 5′-CCUCAAAGCUUCGUGUUAA-3; SMAD4 (EHU018671 esiRNA siRNA mixture, Sigma). A 19-bp scrambled oligomer (sense: 5′-AGGUAGUGUAAUCGCCUUG-3′) (Eurofins MWG Operon, Ebersberg, Germany) was used for mock transfection. Cells were seeded to ~40% confluency in the relevant culture dishes and transfected with NHE1 siRNA (100 nM), NBCn1 siRNA (25 nM), Merlin siRNA (50 nM) or mock siRNA (50 nM), using Lipofectamine (Invitrogen) transfection reagent, according to the manufacturer's specifications. 48 h after transfection, cells were serum starved, and 24 h later, exposed to TGFβ treatment (or corresponding control conditions) for another 48 h before analysis for the relevant experiment as indicated below.

### RT-qPCR Analysis

Total RNA was isolated using NucleoSpin® RNA II (Macherey-Nagel, Germany) according to the manufacturer's instructions, reverse-transcribed using Superscript III Reverse Transcriptase (Invitrogen, Carlsbad, CA) and cDNA transcripts were amplified by qPCR using the SYBR Green technique (Applied Biosystems, Cheshire, UK). Amplification was performed in triplicate in an ABI7900 qPCR machine, using 40 cycles of (95°C for 30 s, 60°C for 1 min, 72°C for 30 s). Primer sequences were: NHE1-fw: 5′-CACACCACCATCAAATACTTCC-3′, NHE1-rv: 5′-GAACTTGTTGATGAACCAGGTC-3′; NHE2-fw: TTGGAGAGTCCCTGCTGAATGATG, NHE2-rv: tcagctgtgatgtaggacaaataactg, NBCn1-fw: 5′-GCAAGAAACATTCTGACCCTCA-3′, NBCn1-rv: 5′-GCTTCCACCACTTCCATTACzCT, NBCe2-fw: atcttcatggaccagcagatcac, NBCe2-rv: tgcttggctggcatcaggaag. mRNA expression was quantified using the comparative threshold cycle (Ct) method, using β-actin as reference gene (fw 5′- AGCGAGCATCCCCCAAAGTT-3′, rv 5′-GGGCACGAAGGCTCATCATT-3′), and is given relative to that in HPDE cells.

### SDS-PAGE and Western Blotting

Cells were seeded to 60–70% confluency in 6-well culture dishes, lysed in lysis buffer [1% SDS, 10 mM Tris-pH 7.5, Na_3_VO_4_ 1 mM, and complete protease inhibitor cocktail (Roche)] and homogenized by sonication. SDS-PAGE was performed using Bio-Rad Criterion^TM^ TGX^TM^ precast 10% gels in Tris/Glycine buffer. Gels were run for 1 h at 150 V, followed by transfer to Trans-Blot® Turbo^TM^ 0.2 μm nitrocellulose membranes (Bio-Rad). Protein transfer was evaluated by Ponceau red staining, followed by blocking in 5% dry milk in TBST (0.01 M Tris/HCl, 0.15 M NaCl, 0.1% Tween 20) for 30 min at room temperature, incubation with primary antibodies overnight at 4°C, and finally washing in TBST and incubation with HRP-conjugated secondary antibodies for 2 h at room temperature. Blots were developed using the FUSION-Fx chemiluminescence system (Vilber Lourmat). Images were processed in Adobe Photoshop and band intensities were quantified using UN-SCAN-IT gel 6.1 software.

### Immunofluorescence Microscopy Analysis

Cells were grown on glass coverslips in 6-well culture dishes, fixed in 4% paraformaldehyde for 15 min at room temperature, washed in icecold PBS, and permeabilized for 12 min in permeabilization buffer (0.2 % Triton X-100, 1% BSA in PBS). Unspecific fluorescence was blocked by a 30 min incubation in PBS plus 2% BSA, followed by incubation with primary antibody for 1$\frac{1}{2}$ h at room temperature, three washes in 2% BSA blocking buffer, and incubation with secondary antibodies diluted in 2% BSA blocking buffer) for 45 min, washing in PBS containing 2% BSA, a 5 min incubation with (4',6-Diamidino-2-Phenylindole, Dihydrochloride) (DAPI) for nuclear staining, extensive washing in PBS and mounting in N-propyl-gallate mounting media (2% w/v in PBS/glycerin). Fluorescence images were captured on a fully motorized Olympus BX63 upright microscope with an Olympus DP72 color, 12.8-megapixel, 4.140 × 3.096-resolution camera and with a fully motorized and automated Olympus IX83 Inverted microscope with a Hamamatsu ORCA-Flash 4.0 camera (C11440-22CU). The software used was Olympus CellSens dimension, which is able to do deconvolution on captured z stacks, and images were processed for publication using Adobe Photoshop CS6.

### Real-Time Imaging of Intracellular pH

Measurements of pH_i_ were carried out essentially as described previously (21). Briefly, cells seeded in WillCo glass-bottom dishes (WillCo Wells, Amsterdam, the Netherlands) were loaded with 2′,7′-bis-(2-carboxyethyl)-5-(and-6)-carboxyfluorescein acetoxymethyl ester (BCECF-AM, 1.6 μM) in growth medium for 30 min at 37°C. Cells were washed once in HCO${}_{3}^{-}$ containing Ringer solution [118 mM NaCl, 25 mM NaHCO_3_, 5 mM KCl, 1 mM MgSO_4_, 1 mM Na_2_HPO_4_, 1 mM CaCl_2_, 3.3 mM 3-(N-morpholino)propanesulfonic acid (MOPS), 3.3 mM 2-[Tris(hydroxymethyl)-methylamino]-ethanesulfonic acid (TES), 5 mM HEPES, pH 7.4], placed in a 37°C imaging chamber equipped with gas and solute perfusion, at the stage of a Nikon Eclipse T*i* microscope, and imaged using a 40X/1.4 NA objective and EasyRatioPro imaging software (PTI, NJ, USA). Emission was measured at 520 nm after excitation at 440 and 485 nm. Acidification was induced by exposure to 20 mM NH_4_Cl for 10 min. Calibration to pH_i_ values was performed using the high K^+^/nigericin technique and a 4-point linear calibration curve.

### BrdU Proliferation Assay

Eighty percentage confluent Panc-1 and BxPC3 cells were trypsinized and resuspended in growth medium. Cells were seeded in 96-well plates (Celllstar®, cat # 655090). After 24 h incubation at 37°C/5% CO_2_, plates were washed in PBS and 200 μl serum free medium was added. After 24 h, the cells were incubated with 10 ng/mL TGFβ, and/or 10 μM of the NHE1 inhibitor cariporide. Forty-eight hours later, a Cell Proliferation ELISA, BrdU (chemiluminescent) kit (Roche, cat # 11 669 915 001) was used for determining the proliferation status of the cells, according to the manufacturer's instructions. 20 μl BrdU labeling solution was added to each well and the plates were incubated for 4 h, followed by a fixation/denaturing step, and incubation with a peroxidase-coupled mouse monoclonal anti-BrdU antibody. The plates were washed extensively, incubated with substrate solution containing luminol, 4-iodophenol and peroxide for 4 min on a shaker, and luminescence measured using a BMG FLUOstar OPTIMA Microplate Reader.

### Invasion Assay

Cell invasion was assessed using growth factor reduced corning® Matrigel® invasion chambers, 24-well plate and 8.0 μm pore size (Corning, BioCoat, MA, USA). Prior to the experiment, invasion chambers containing Matrigel were rehydrated by adding 500 μl serum free medium for 2 h at 37°C. Forty-eight hours after transfection with the indicated siRNAs, cells were serum starved, and 24 h later, treated or not with TGFβ for 48 h, before being washed with sterile PBS, trypsinized, washed 3 x in serum free medium, and 50,000 cells in this medium seeded into the upper chamber. Experiments were always done in duplicate. The lower chamber was filled with 10% serum containing medium. Chambers were incubated for 22 h at 37°C/5% CO_2_ to allow cells to invade through the Matrigel. Non-invaded cells from the upper surface of the chamber were removed with a cotton swab. Invaded cells on the lower surface of the chamber were fixed for 30 min in ice-cold absolute methanol, and filters were stained with 30% Giemsa solution (Sigma-Aldrich) for 30 min and mounted on glass slides. Invaded cells (four images per filter per condition) were counted using bright-field microscopy.

### Data Analysis and Statistics

Data are shown as individual representative experiments or as means of at least three independent experiments, with standard error of mean (SEM) error bars. Statistical significance was tested using Student *t*-test or one- or two-way ANOVA followed by Tukey's multiple comparison tests as appropriate, using GraphPad prism 6.

## Results

### Expression of Net Acid Extruding Transporters Is Increased in PDAC Cells

**Figure 1:**
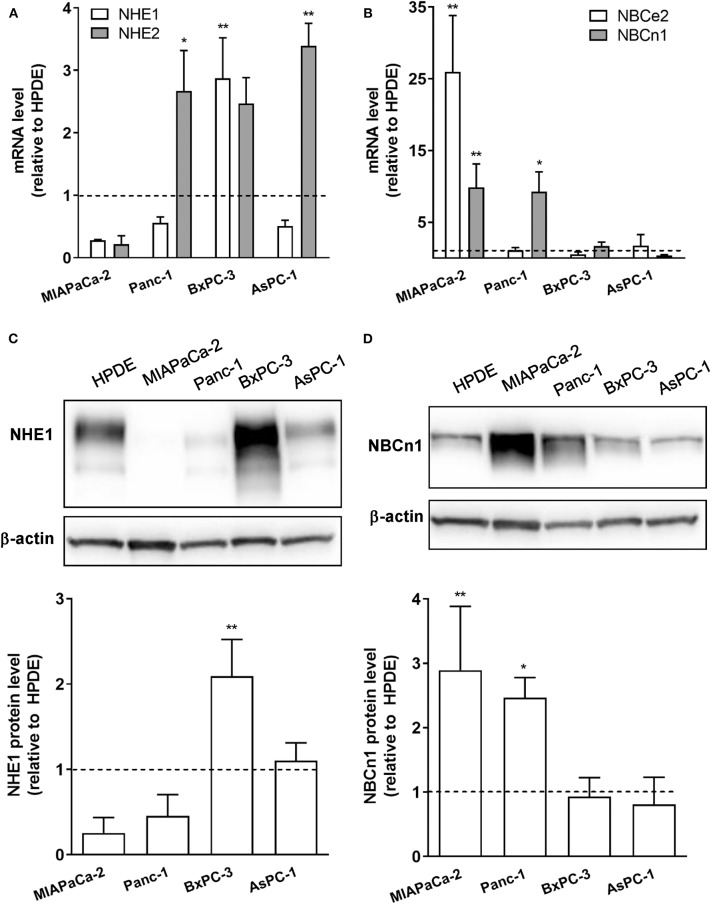
mRNA and protein expression of net acid extruding transporters is upregulated in PDAC cells compared to normal pancreatic ductal epithelial cells. **(A,B)** Immortalized pancreatic ductal epithelial (HPDE) cells and a panel of PDAC cell lines: MIAPaCa-2, Panc-1, BxPC-3, and AsPC-1 cells, were grown to about 80% confluence, lysed and mRNA expression of NHE1 and−2 **(A)** and NBCe2 and NBCn1 **(B)** analyzed by RT-qPCR. Data (mean with S.E.M. error bars) are normalized to the expression level in HPDE cells in the same experiment (shown as a dotted line). **(C,D)** HPDE, MIAPaCa-2, Panc-1, BxPC-3, and AsPC-1 cells were grown to about 80% confluence, lysed and protein levels of NHE1 **(C)** and NBCn1 **(D)** analyzed by Western blotting. Data (mean with S.E.M. error bars) are normalized to the β-actin loading control and to the expression level in HPDE cells in the same experiment (shown as a dotted line). *n* = 3–4 independent experiments per condition in all panels. ^*^, ^**^ One-way ANOVA, *p* < 0.05 and 0.01, respectively, against the level in HPDE cells.

Our previous *in silico* analysis of RNA sequencing data from pancreatic cancer patient tissue indicated that a number of SLC9 and SLC4 family transporters are upregulated in PDAC patient tumor tissue compared to normal pancreatic epithelium (29). From these, we selected for widely expressed net acid extruders for initial analyses: two Na^+^/H^+^ exchangers, NHE1 and−2 (SLC9A1-2), and two Na^+^,HCO${}_{3}^{-}$ cotransporters (NBCs), NBCe2 (SLC4A5) and NBCn1 (SLC4A7). The relative mRNA levels of the transporters were determined by RT-qPCR analysis in immortalized pancreatic epithelial (HPDE) cells (27, 28), and in a panel of human PDAC cell lines of different genotypes: MIAPaCa-2, Panc-1, BxPC-3, and AsPC-1 cells. All PDAC cell lines studied exhibited increased mRNA levels of one or more of these transporters compared to HPDE cells, with the specific transporters upregulated differing between cell lines (Figures 1A,B). Thus, BxPC-3 and AsPC-1 cells showed elevated expression of NHE1 and−2, and MIAPaCa-2 cells predominantly showed upregulation of NBCn1 and NBCe2, while both NHE2 and NBCn1 were upregulated in Panc-1 cells. For further analyses, we focused on NHE1 and NBCn1, which play central roles in the development of PDAC and other cancers (21, 22, 24, 30–33). Consistent with the mRNA data, western blot analysis revealed that the NHE1 protein level was significantly increased in BxPC-3 cells and the NBCn1 protein level in MIAPaCa-2 and Panc-1 cells, compared to that observed in HPDE cells (Figures 1C,D).

These data show that net acid extruding proteins are upregulated in PDAC cells compared to normal pancreatic ductal epithelial cells, and that the specific pattern of upregulation is cell type dependent.

### TGFβ-1 Treatment Elicits a SMAD4-Dependent EMT in PDAC Cells

**Figure 2:**
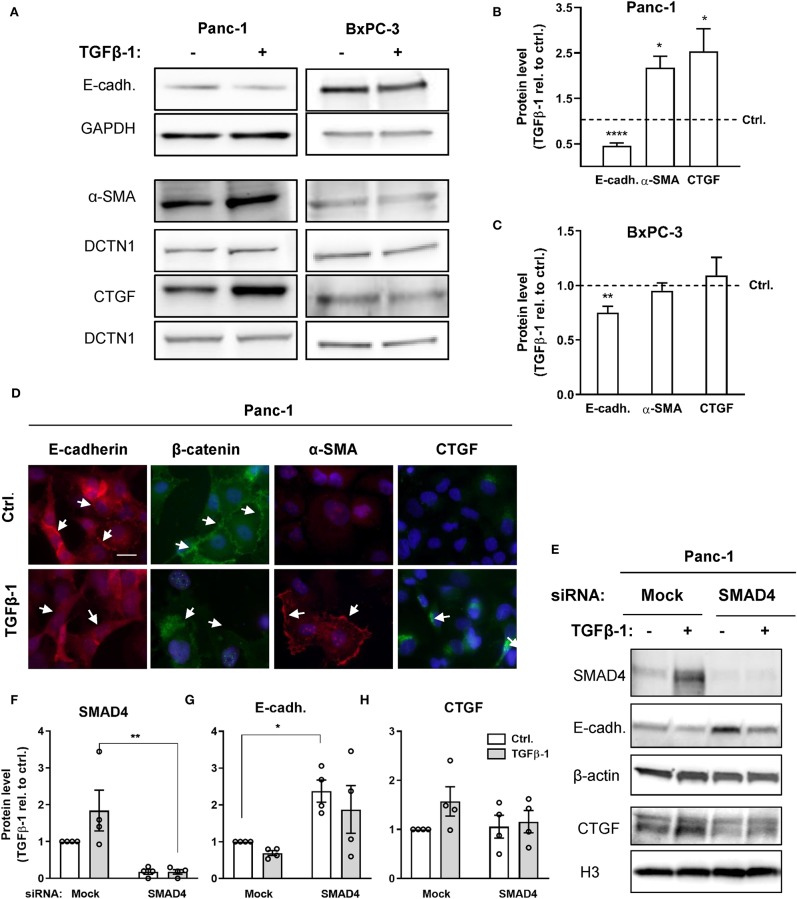
TGFβ-induces a predominantly SMAD4-dependent EMT. **(A)** Panc-1 and BxPC-3 cells were serum starved for 24 h, followed by 48 h growth with or without 10 ng/ml human recombinant TGFβ-1 for 48 h as indicated. Cells were lysed and subjected to western blotting for E-cadherin, α-SMA, and CTGF, using GAPDH and DCTN1 as loading markers. Representative western blots are shown. **(B,C)** Summarized, quantified data from experiments as in **(A)**, for Panc-1 cells **(B)** and BxPC-3 cells **(C)**. Data (mean with S.E.M. error bars) were normalized to loading control and to the level in the absence of TGFβ-1 (Ctrl.), shown by the dotted line. *n* = 3–8 independent experiments per condition. *, **, **** One-way ANOVA, *p* < 0.05, 0.01, 0.0001, respectively, against the level in non-TGFβ-1 treated control cells. **(D)** Immunofluorescence microscopy analysis of the localization and expression level of E-cadherin, β-catenin, α-SMA and CTGF in Panc-1 cells. Nuclei are stained using DAPI. The images shown are representative of at least three independent experiments per condition. Arrows indicate the distinct plasma membrane localization of E-cadherin and β-catenin under control conditions and the shift in localization upon TGFβ-1 treatment. **(E–H)** Effect of SMAD4 knockdown in Panc-1 cells on E-cadherin and CTGF protein levels. E, representative western blot, F-H, SMAD4, E-cadherin, and CTGF protein levels, normalized to mock ctrl. and shown as mean with S.E.M. error bars and individual data points. Relative to mock siRNA-treated controls, SMAD4 expression was 1.8 ± 0.55 in mock siRNA + TGFβ-1 treated cells, 0.18 ± 0.066 in SMAD4 siRNA treated cells, and 0.18 ± 0.063 in SMAD4 siRNA + TGFβ-1 treated cells. β-actin and Histone 3 (H3) are used as loading markers. Data are from four independent experiments per condition. *, ** One-way ANOVA, *p* < 0.05, 0.01, respectively.

BxPC-3 and Panc-1 cells, which display a high levels of NHE1 and NBCn1 expression, respectively, were chosen for further analysis. We first assessed the ability of TGFβ ligand to induce EMT in the two cell lines. Cells were serum-starved for 24 h, followed by treatment with 10 ng/ml human recombinant TGFβ-1 for 48 h. After lysis, cells were subjected to western blot analysis for the epithelial marker E-cadherin as well as the mesenchymal markers α-smooth muscle actin (α-SMA) and connective tissue growth factor (CTGF) (Figure 2A). In Panc-1 cells, TGFβ-1 treatment significantly reduced the level of E-cadherin, and increased that of α-SMA and CTGF, consistent with induction of EMT (Figures 2A,B). In BxPC-3 cells, TGFβ-1 treatment elicited a small yet significant decrease in E-cadherin of about 25%, which was not accompanied by detectable changes in α-SMA and CTGF expression (Figures 2A,C). Immunofluorescence analysis was performed to assess the impact of TGFβ-1 treatment on cell morphology and protein localization in Panc-1 cells. TGFβ-1 treatment induced a marked internalization of both E-cadherin and β-catenin (arrows), a strong, predominantly membrane-associated increase in α-SMA, and increased intracellular CTGF expression (Figure 2D).

These data show that TGFβ-1 treatment elicited a marked EMT in Panc-1 cells, and a modest and partial EMT in BxPC-3 cells.

BxPC-3 cells are SMAD4-deficient (34), which has previously been shown to be responsible for their partial resistance to TGFβ-1-induced EMT (14), although the absolute requirement for SMAD4 for TGFβ-1-induced EMT is controversial (14, 35). To assess the role of SMAD4 in TGFβ-1-induced EMT in Panc-1 cells, we knocked down SMAD4 by siRNA in Panc-1 cells, followed by TGFβ-1 treatment for 48 h, and western blotting for E-cadherin and CTGF (Figures 2E–H). In mock-transfected cells, TGFβ-1 treatment almost doubled the level of SMAD4. Confirming efficient knockdown, the SMAD4 protein level was reduced by more than 80% in SMAD4 siRNA-treated cells (Figures 2E,F). Notably, the E-cadherin level was more than doubled by SMAD4 knockdown in the absence of TGFβ-1, and this was only marginally reduced by TGFβ-1-induced increase in CTGF expression was abolished in SMAD4-depleted cells (Figure 2G). Conversely, SMAD4 knockdown had no effect on the CTGF level under control conditions yet abolished the increase in CTGF induced by TGFβ-1 treatment (Figure 2H).

Collectively, these data show that TGFβ-1 induced EMT in Panc-1 cells is strongly dependent on SMAD4 and suggest that a basal level of SMAD4 signaling in the absence of TGFβ-1 treatment is responsible for the low basal E-cadherin level in these cells.

### NHE1 Expression Is Increased in a SMAD-Dependent Manner During TGFβ-1-Induced EMT

**Figure 3:**
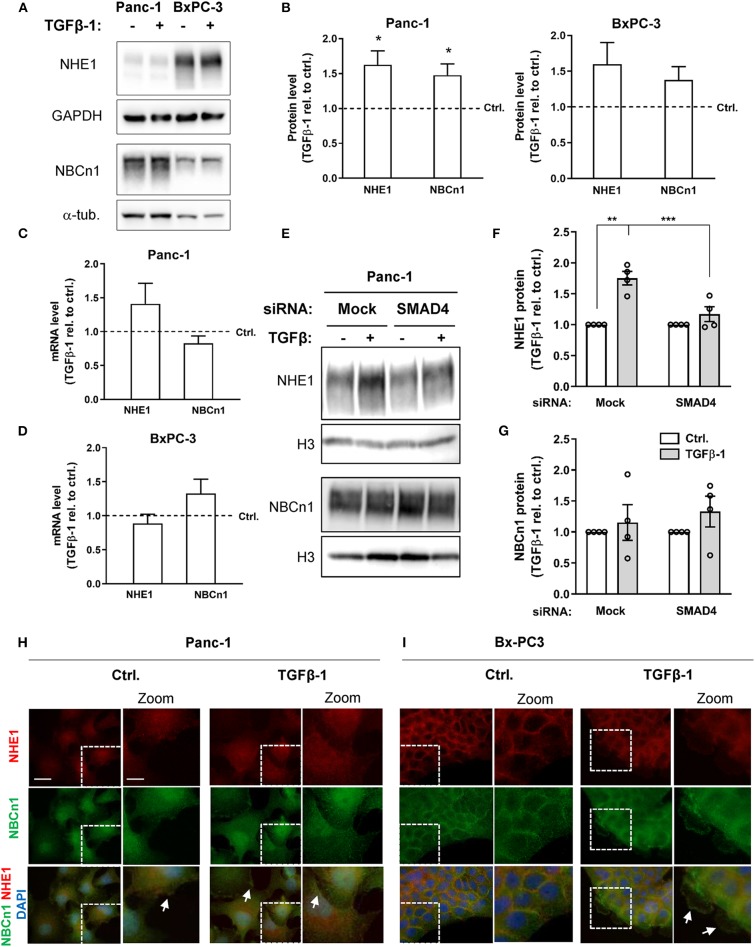
NHE1 and NBCn1 protein expression is upregulated during TGFβ-induced EMT. **(A,B)** Following growth in absence or presence of 10 ng/ml human recombinant TGFβ-1 for 48 h, cells were lysed and subjected to western blotting for NHE1 and NBCn1. **(A)** representative blots, with GAPDH and α-tubulin as loading markers. **(B)** quantified data for Panc-1 and BxPC-3 cells, shown as mean with S.E.M. error bars, normalized to the level in absence of TGFβ-1 (represented by the dotted line). Data shown represent *n* = 3–8 per condition. * One-way ANOVA, *p* < 0.05, against the level in non-TGF-β treated control cells. **(C,D)** Panc-1 **(C)** and BxPC-3 **(D)** cells were serum-starved for 24 h, followed by TGFβ-1 treatment as above for 48 h. mRNA levels were determined by qPCR and normalized to non-TGF-β treated control cells. Data are mean with S.E.M. error bars, of 8-11 independent experiments per condition. **(E–G)** Effect of SMAD4 knockdown on NHE1 and NBCn1 protein levels in Panc-1 cells. **(E)** representative western blot, **(F,G)** NHE1 and NBCn1 protein levels, normalized to mock ctrl. and shown as mean with S.E.M. error bars and individual data points. Data shown are from 4 independent experiments per condition. For SMAD4 knockdown efficiency, see Figure 2E and Figure 2 legend. **, *** One-way ANOVA, *p* < 0.05, 0.01, respectively. **(H,I)** IFM analysis illustrating the localization and expression level of native NHE1 (red) and NBCn1 (green) in Panc-1 **(H)** and BxPC-3 **(I)** cells. Nuclei are stained using DAPI. Images shown represent at least three independent experiments per condition. Scale bars are 20 μm, and 10 μm in the zoomed images.

We next asked whether TGFβ-1 treatment altered NHE1 and NBCn1 expression in Panc-1 and BxPC-3 cells. The protein expression level of NHE1 increased by about 60%, and that of NBCn1 by about 40%, following TGFβ-1 treatment of Panc-1 cells (Figures 3A,B), and a similar, albeit non-significant trend was seen in BxPC-3 cells (Figures 3A,C). The mRNA level of NHE1 also tended to be upregulated by TGFβ-1 in Panc-1 cells, and that of NBCn1 in BxPC-3 cells (Figures 3C,D). The TGFβ-1-induced increase in NHE1 expression was strongly reduced by SMAD4 knockdown in Panc-1 cells (Figures 3E,F), whereas the modest increase in NBCn1 expression was not affected by SMAD4 knockdown (Figures 3E,G). This suggests that increased NHE1 expression is mainly mediated by canonical TGFβ signaling, whereas the effect of TGFβ-1 on NBCn1 expression appears to be mediated by non-canonical signaling events. Both transporters localize predominantly to the plasma membrane in both cell types. Interestingly, TGFβ-1 treatment caused a characteristic redistribution of NBCn1 to membrane ruffles in BxPC-3 cells (Figure 3I) whereas there was no detectable redistribution in Panc-1 cells (Figure 3H).

To determine whether NHE1 and NBCn1 played a role in the EMT process *per se*, we knocked down each transporter in Panc-1 cells and subjected the cells to 48 h of TGFβ-1 treatment as above, followed by western blotting for E-cadherin and CTGF. Although the protein level of both transporters was essentially abolished upon siRNA treatment, this did not detectably affect TGFβ-1–induced E-cadherin downregulation or CTGF upregulation (Supplementary Figure 1).

These results show that TGFβ-1 induced EMT is associated with increased expression of NHE1 and NBCn1 and increased localization of the transporters at the plasma membrane where they could increase net acid extrusion from the cells. However, neither transporter is required for induction of EMT *per se*.

### TGFβ-1 Increases Steady State pH_i_ and NHE1-Dependent Acid Extrusion in Panc-1 Cells

**Figure 4:**
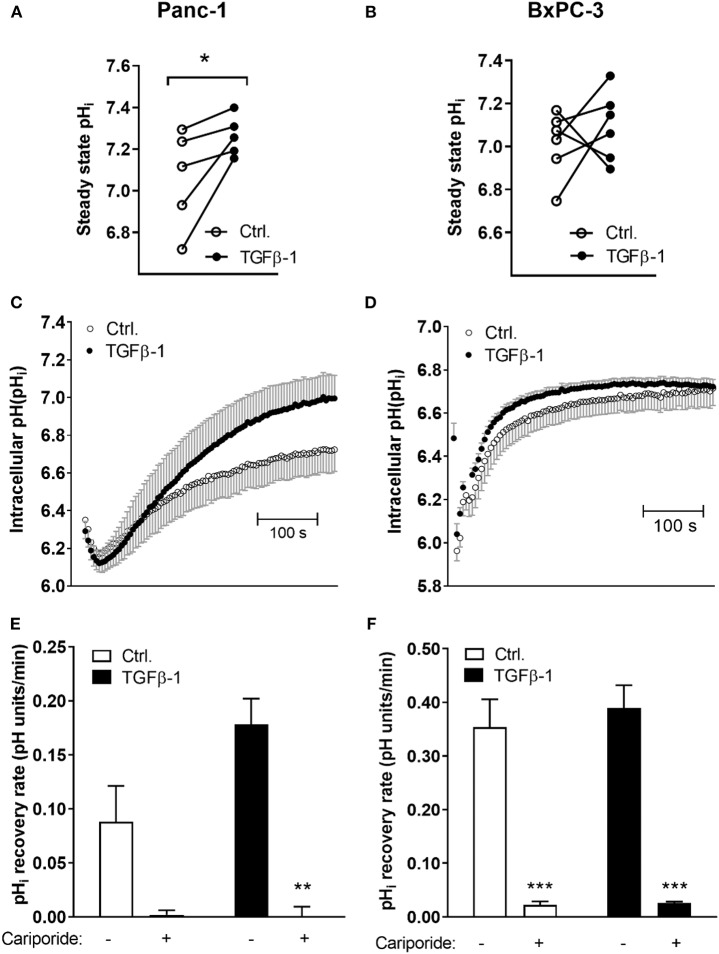
TGFβ-1 increases steady state pH_i_ and NHE1-dependent acid extrusion in Panc-1 cells. Cells were treated ±10 ng/ml TGFβ-1 for 48 h, and intracellular pH (pH_i_) was monitored by real-time imaging analysis in Panc-1 **(A,C,E)** and BxPC-3 **(B,D,F)** cells after loading of cells with BCECF-AM. Experiments were conducted at 37°C, and under CO_2_/HCO${}_{3}^{-}$ buffered conditions. **(A,B)** Steady state pH_i_ was increased by TGFβ-1 in Panc-1 but not in BxPC-3 cells. The graphs show the pH_i_ in paired control and TGFβ-1 treated cells from each experiment. The mean pH_i_ values were, for Panc-1 cells, 7.06 ± 0.105 in control cells and 7.26 ± 0.043 after TGFβ-1 treatment, and in BxPC-3 cells, 7.01 ± 0.062 in controls and 7.09 ± 0.066 after TGFβ-1. Data are based on 5 (Panc-1) and 6 (BxPC-3) independent biological experiments for each condition. * significantly different, *p* < 0.05, paired *t*-test. **(C–F)** The pH_i_ recovery rate was determined as the initial rate of recovery after NH_4_Cl-prepulse-induced intracellular acidification. Where indicated, cariporide (10 μM) was present during the recovery phase to inhibit NHE1. **(C,D)** Representative traces with SD error bars, **(E,F)** means with S.E.M. error bars of 3–5 independent experiments per condition. **, *** Paired *t*-test, *p* < 0.01 and 0.001, respectively against corresponding conditions (Ctrl, TGFβ) in absence of cariporide).

To directly determine whether the changes in transporter expression upon TGFβ-1-induced EMT are associated with altered pH_i_ homeostasis, cells were subjected to TGFβ-1 treatment for 48 h, loaded with the pH_i_ sensitive fluorophore BCECF-AM, and steady state pHi determined by live imaging of BCECF fluorescence. Experiments were carried out in the presence of CO_2_/HCO${}_{3}^{-}$ to allow contributions from bicarbonate-dependent transporters such as NBCn1. TGFβ-1 treatment significantly increased steady state pH_i_ in Panc-1 cells, from an average value of 7.06 ± 0.105 in control cells to 7.26 ± 0.043 after TGFβ-1 treatment (Figure 4A). In contrast, the steady state pH_i_ of BxPC-3 cells was 7.01 ± 0.062 in controls and 7.09 ± 0.066 after TGFβ-1 treatment, not significantly different (Figure 4B).

To evaluate whether cellular capacity for acid extrusion was increased by TGFβ-1 treatment, we next determined the pH_i_ recovery rate. Cells were pretreated with or without TGFβ-1 for 48 h as above, and subjected to an NH_4_Cl-prepulse to acidify the cells: after equilibration in normal CO_2_/HCO${}_{3}^{-}$ saline, cells were perfused with 15 mM NH_4_Cl. Upon dissociation, NH_3_ rapidly enters the cells by diffusion. Its association with cellular H^+^ to NH${}_{4}^{+}$ causes near-instantaneous alkalinization, followed by slow return toward steady state pH_i_ as NH${}_{4}^{+}$ from the solution enters the cells via ion transporters, shifting the equilibrium. When cells are again perfused with normal CO_2_/HCO${}_{3}^{-}$ saline, all NH_3_ rapidly diffuses out, and an excess of free H^+^ is left in the cells. The rate of recovery from this acidification is a measure of net acid extrusion capacity, determined as the slope of the initial, linear part of the curve under conditions where starting pH_i_ is similar (36). Experiments were again carried out in the presence of CO_2_/HCO${}_{3}^{-}$. Figures 4C,D shows representative traces of pH_i_ over time from the maximal acidification. As seen, TGFβ-1 treatment markedly increased the pH_i_ recovery rate in Panc-1 cells (Figure 4C) but not in BxPC-3 cells (Figure 4D). NHE1 and NBCn1 activity is posttranslationally regulated (37, 38), hence, their contributions to pH_i_ regulation are not predicted by their protein levels. Notably, in both Panc-1 and BxPC-3 cells, pH_i_ recovery in both control- and TGFβ-1 treated cells was abolished by the specific NHE1 inhibitor cariporide regardless of (Figures 4E,F).

Collectively, these results show that TGFβ-1-induced EMT in Panc-1 cells is associated with increased steady state pH_i_ and increased NHE1-dependent acid extrusion capacity.

### TGFβ-1 Differentially Regulates Cell Proliferation in Panc-1 and BxPC-3 Cells

**Figure 5:**
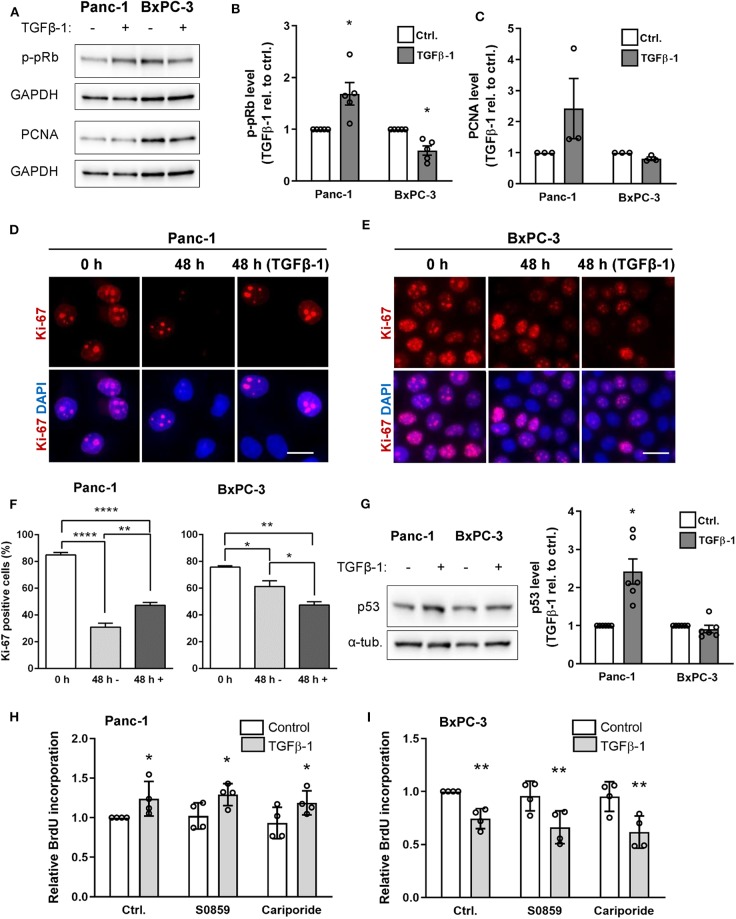
TGFβ-1 stimulates proliferation in Panc-1 cells, and inhibits it in BxPC-3 cells. **(A)** Panc-1 and BxPC-3 cells were serum starved for 24 h, followed by 48 h growth with or without TGFβ-1 as indicated. Cells were lysed and blotted for p-pRb and PCNA, using GAPDH as loading marker. **(B,C)** Relative p-pRB and PCNA levels from data as in **(A)**. Data are normalized to ctrl. and shown as mean with S.E.M. error bars and individual data points. *n* = 3–5 per condition. *p < 0.05, paired, two-tailed *t*-test, against the level in non-TGFβ-1 treated cells. **(D,E)** Immunofluorescence analysis of Ki-67 after 0 or 48 h of serum starvation, the latter ±TGFβ-1 as shown. Ki-67 positive cells are quantified in **(F)**. Data represent 3 independent experiments. **(G)** Panc-1 and BxPC-3 cells were serum starved for 24 h, grown for 48 h with or without TGFβ, lysed and blotted for p53. *n* = 4. **(H,I)** Proliferation of Panc-1 **(H)** and BxPC-3 **(I)** cells determined by BrdU incorporation, in absence or presence of NHE1 inhibitor cariporide (10 μM) or NBC inhibitor S0859 (10 μM). *n* = 4. Data are normalized to untreated ctrl. and shown as mean with S.E.M. error bars and individual data points. *p < 0.05, ** *p* < 0.01, and **** *p* < 0.0001, respectively.

Depending on the cell type and context, TGFβ signaling can counteract or stimulate cell proliferation, and this has been ascribed at least in part to SMAD2/3 signaling, which rely on SMAD4 (4, 6). We therefore assessed the impact of TGFβ-1 treatment on cell proliferation in Panc-1 and BxPC-3 cells. Cells treated for 48 h with or without TGFβ-1 were lysed and blotted for phosphorylated retinoblastoma protein (p-pRb) and proliferating cell nuclear antigen (PCNA) as markers of cell cycle entry and progression, respectively. Notably, TGFβ-1 treatment increased the p-pRb level in Panc-1 cells yet decreased it in BxPC-3 cells (Figures 5A,B), and similar results were obtained for PCNA (Figures 5A,C). This was confirmed by IFM analysis using Ki-67 as a proliferation marker: The fraction of Ki-67 positive cells was decreased in both cell types—although most dramatically in Panc-1 cells—by 48 h of serum starvation, and was increased in Panc-1 cells, yet decreased in BxPC-3 cells by TGFβ-1 (Figures 5D–F). Strikingly, TGFβ-1 treatment also increased p53 expression in Panc-1 cells, but not in BxPC-3 cells (Figure 5G). Co-staining for p53 and p-pRb confirmed these data and revealed that elevation of nuclear staining for p-pRb and p53 was detected within the same cells (Supplementary Figure 2). BrdU incorporation analysis confirmed that TGFβ-1 treatment increased proliferation of Panc-1 cells, yet decreased that of BxPC-3 cells, and showed that proliferation was unaffected by inhibition of NHE1 (10 μM cariporide) or NBCs (10 μM S0859), under both basal and TGFβ-1-treated conditions (Figures 5H,I).

Taken together, these results show that TGFβ-1 treatment stimulates proliferation of Panc-1 cells yet inhibits that of BxPC-3 cells. This occurs in parallel with a TGFβ-1-induced increase in p53 expression in the Panc-1 cells and is unaffected by inhibition of NHE1 or NBCn1.

### Loss of Merlin Stimulates TGFβ-1-Induced Invasiveness in an NHE1- and NBCn1-Dependent Manner

Previous studies have demonstrated the involvement of acid-base transporters in cell motility and invasiveness, through roles of the transporters in cell adhesion, cytoskeletal dynamics,and matrix degradation (19, 25, 39–41). We therefore asked whether the TGFβ-1-induced upregulation of NHE1 and NBCn1 contributed to TGFβ-1-induced invasiveness of Panc-1 cells.

**Figure 6:**
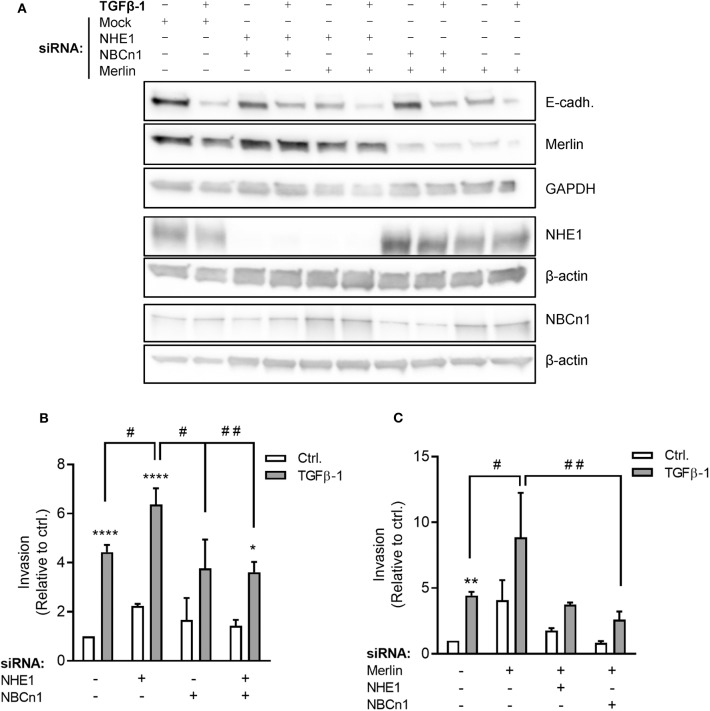
Roles of NHE1, NBCn1, and Merlin in TGFβ-induced invasiveness of Panc-1 cells. **(A)** Panc-1 cells were treated with siRNA against NHE1, NBCn1, and /or Merlin as indicated, starved for 48 h, and treated or not with TGFβ-1 for 48 h, followed by Western blotting to assess protein expression levels. Data represent three independent experiments. **(B)** Panc-1 cells were treated with siRNA against NHE1 or NBCn1 as indicated, treated or not with TGFβ-1 as above for 48 h, and seeded at 50,000 cells/well in in serum-free medium in Matrigel-coated Boyden chambers. The lower chamber contained 10% FBS. Twenty-two hours later, experiments were terminated and the number of invaded cells determined. **(C)** As **(B)**, except including knockdown of merlin as indicated. *n* = 3–15 independent experiments, each done in duplicate, per condition. *, **, ****) *p* < 0.05, 0.01, and 0.0001, respectively, 2-way ANOVA, ctrl. vs. TGFβ; #, ## *p* < 0.05, 0.01, respectively, 2-way ANOVA between the indicated conditions.

Stimulation with TGFβ-1 increased invasion of Panc-1 cells through Matrigel more than 4-fold (Figure 6B), consistent with the strong TGFβ-1-induced EMT induction in these cells (Figure 2). siRNA-mediated knockdown of NHE1 further increased TGFβ-1-induced invasiveness, whereas knockdown of NBCn1 or both transporters in combination had no effect (Figures 6A,B). The unexpected exacerbation of invasiveness by NHE1 knockdown prompted us to ask whether the impact of NHE1 on PDAC cell invasiveness was genotype-dependent. The tumor suppressor Merlin (aka Neurofibromatosis type 2, NF2), which is downregulated in many PDAC tumors (42), was reported to regulate EMT (43) and cell motility (44), and was recently shown to regulate NHE1 in melanoma cells (45). siRNA-mediated knockdown of Merlin (Figure 6A) increased basal invasion 4-fold and almost doubled TGFβ-1-induced invasiveness (Figure 6C). Notably, in Merlin-depleted cells, knockdown of either NHE1 or NBCn1 abolished the increase in invasion (Figure 6C).

These results show that Merlin depletion increases basal invasion and potentiated TGFβ-1-induced invasiveness. Invasion induced by Merlin depletion, but not that induced by TGFβ-1 alone, is dependent on NHE1 and NBCn1.

## Discussion

Pancreatic cancer has one of the most ominous mortality rates of any cancer globally, and the relative burden of disease is expected to increase over the next decade (1). PDAC tumors frequently exhibit abundant secretion of TGFβ from both stromal and tumor cells (46). Another characteristic of PDAC is extensive EMT (14, 15), the roles of which in PDAC development are incompletely understood (11, 16).

An emerging hallmark of solid tumors is profound dysregulation of pH homeostasis and upregulation of net acid-extruding transport proteins which play important roles in cancer development (19, 20). Given the extreme acid-base homeostasis of the pancreas under physiological conditions (3), PDAC is particularly interesting in this context. Here, we asked whether TGFβ-1-induced EMT in PDAC cells is associated with upregulation of net acid extruding transporters. We focused on NHE1 and NBCn1, which we and others have shown to play important roles in cancer development (21–24), and found that these transporters were upregulated in PDAC cell lines compared to normal controls. The great majority of patient PDAC tumors show activating KRAS mutations, and about 50% show mutations in TGFβ pathway components, most commonly SMAD4 loss or inactivating mutations (4). For further analysis we therefore selected Panc-1 cells, which harbor an activating KRAS mutation and express wild type SMAD4, and BxPC-3 cells, which are KRAS wild type and express a truncated, defective version of SMAD4 (34). Consistent with previous reports (14, 15, 35), 48 h TGFβ-1 treatment robustly induced EMT characteristics, i.e., downregulation of E-cadherin, upregulation of α-SMA and CTGF, and internalization of β-catenin in Panc-1 cells, compared to a partial EMT in BxPC-3 cells where only E-cadherin expression was detectably altered. Accordingly, SMAD4 knockdown in Panc-1 cells largely abolished EMT. EMT has been reported to involve both SMAD4-dependent (17) and–independent (18) processes. TGFβ-induced E-cadherin downregulation in Panc-1 cells and other PDAC cell lines was reported to be nearly completely dependent on SMAD3 and SMAD4, as well as the EMT-associated transcription factors SLUG and SNAIL, which are activated downstream from SMAD4 (35). The SMAD3-4 complex binds and activates the SNAIL promoter, in turn leading to the SNAIL-dependent E-cadherin downregulation (47). However, non-canonical TGFβ pathways including ERK1/2 were also reported to contribute to EMT (35), consistent with our finding of partial EMT induction in BxPC-3 cells (34).

A central finding of this work was that TGFβ-1-induced EMT was associated with upregulation of NHE1, and to a lesser extent NBCn1, expression in Panc-1 cells. Consistent with this, NHE1 was recently proposed to be upregulated by ZEB1, a transcription factor involved in driving EMT (48). Knockdown of the NHE1 or NBCn1 had no detectable effect on EMT induction *per se* (E-cadherin, CTGF and α-SMA levels). However, in congruence with the transporter upregulation, EMT induction was associated with an increase in steady state pH_i_ and increased capacity for net acid extrusion in Panc-1 cells but not in BxPC-3 cells. Acid extrusion was potently inhibited by the NHE1 inhibitor Cariporide in both cell lines, despite the presence of CO_2_/HCO${}_{3}^{-}$ and the relatively high expression of NBCn1 in the Panc-1 cells. Both cell lines also express NHE2 at rather high levels (Figure 1A). With reported Ki values for Cariporide of 0.05 μM for NHE1 and 3 μM for NHE2 in transfected fibroblasts (49), it seems likely that both isoforms might contribute to this recovery (see also below). Notably, the stimulatory effect of TGFβ on pH_i_ regulation in Panc-1 cells differs from the reported inhibitory effect of TGFβ on pH_i_ regulation in non-cancer hepatocytes (50). Thus, it appears that, fully in line with the opposite effects of TGFβ signaling on proliferation and survival in cancer- and non-cancer cells (6, 8), TGFβ attenuates pH_i_ regulation in normal cells, yet stimulates it in some cancer cells.

TGFβ-1 treatment significantly increased proliferation of the SMAD4-positive Panc-1 cells, while decreasing it in the SMAD4-deficient BxPC-3 cells. Paradoxically, at the same time, TGFβ treatment increased p53 expression in Panc-1 cells but not in BxPC-3 cells. Thus, clearly, the presence of SMAD4 and TGFβ-1-induced p53 signaling does not prevent a pro-proliferative effect of the ligand. Both TGFβ and acid extruding proteins are important regulators of cell proliferation/death balance, and EMT has recently been recognized to play important roles also in control of cancer cell survival (11, 16). We therefore reasoned that transporter upregulation might regulate the balance between pro-death and pro-growth effects of TGFβ in PDAC, yet, inhibition of NHE1 or NBCs did not affect proliferation of either cell line under these conditions. It remains possible that such effects might be uncovered in the severely nutrient-deprived and hypoxic tumor microenvironment, where NHE1 has been proposed to play a role in nutrient uptake in PDAC cells via macropinocytosis (51).

Given the roles of pH_i_ and pH_e_ in general, and NHE1 and NBCn1 in particular, in cancer cell motility and invasion (19, 25, 39–41), we asked whether NHE1 and NBCn1 impacted TGFβ-1-induced invasiveness. Forty-eight hours of TGFβ-1 treatment robustly increased invasiveness of Panc-1 cells and this was not abolished by transporter knockdown; in fact, siRNA-mediated depletion of NHE1 modestly increased invasiveness, a finding reminiscent of our demonstration that counter to its general role in favoring motility and invasiveness (25), NHE1 inhibition actually increased motility of p95HER-overexpressing breast cancer cells, while inhibition of NBCs had no effect (52). The lack of effect of NHE1 knockdown shown here contrasts the conclusion of a recent report using 10 μM cariporide to inhibit NHE1 (32). Since NHE1 knockdown was highly efficient in our study, it seems possible that the effect of cariporide may reflect a contribution of NHE2, which, as noted above, would also have been inhibited at this concentration. The tumor suppressor Merlin exhibits reduced expression in PDAC patients (42, 53), is regulated by TGFβ (54), has been assigned a role in regulation of EMT in ARPE-19 cells (43), and in regulation of NHE1 in melanoma cells (45). We therefore hypothesized that Merlin depletion would impact TGFβ-1-induced invasiveness and its regulation by NHE1 and NBCn1. Indeed, Merlin knockdown in itself robustly increased invasiveness, and under these conditions, knockdown of either NHE1 or NBCn1 decreased invasiveness, a tendency seen under both basal and TGFβ-stimulated conditions. This indicates that the roles of NHE1 and NBCn1 may be particularly important in cancers with Merlin downregulation. It is well documented that the roles of pH regulatory transporters in invasion involve effects on focal adhesion strength and turnover, cytoskeletal dynamics, and matrix degradation, downstream of transporter-mediated changes in pH_i_ and pericellular pH_e_ (41). While the precise roles of the transporters in Panc-1 cell invasion were not further studied here, this is supported by the increased pH_i_ and acid extrusion capacity in TGFβ-stimulated Panc-1 cells also demonstrated here.

A limitation of this study is that we did not study the correlation of NHE1 and NBCn1 with EMT markers, TGFβ-signaling pathway components, and Merlin expression in patient tumor tissue from primary pancreatic tumors and metastases. Future work should address such correlations to evaluate the relevance of TGFβ-mediated regulation of these transporters to invasiveness in patients.

In conclusion, we show here that NHE1 and NBCn1 expression and NHE-dependent acid extrusion are upregulated during TGFβ-1-induced EMT of Panc-1 cells. NHE1 upregulation is SMAD4-dependent, and SMAD4-deficient BxPC-3 cells show no change in pH_i_ regulation. The difference between Panc-1 and BxPC-3 cells is corroborated in opposite effects of TGFβ-1 on cell proliferation, which is increased in Panc-1 and decreased in BxPC-3 cells by treatment with this ligand. Knockdown of Merlin strongly potentiates TGFβ-1-induced Panc-1 cell invasiveness in a manner dependent on acid-extruding transporters. We suggest that these transporters are novel players in the events induced during TGFβ-1-induced EMT in PDAC cells.

## Data Availability Statement

The datasets generated for this study are available on request to the corresponding author.

## Author Contributions

SP, SC, and LP developed the concept. SP, SC, RM, and KZ designed the experiments. RM, PS, and KZ performed the experiments for Figures 2A–D, 3A–D,H–I, and RM the experiments for Figure 6 and Supplementary Figure 1. SP performed the experiments in Figure 4. Experiments in Figures 5A–G and Supplementary Figure 2 were performed by PS, and Figures 5H–I by ML. Data analysis and figures were done by SP, RM, KZ, PS, and ML. RM and SP wrote the manuscript with comments and inputs from all co-authors. All authors have seen and approved the final version of the manuscript.

## Conflict of Interest

The authors declare that the research was conducted in the absence of any commercial or financial relationships that could be construed as a potential conflict of interest.
