# Supplementary material for: TGFβ Signaling Increases Net Acid Extrusion, Proliferation and Invasion in Panc-1 Pancreatic Cancer Cells: SMAD4 Dependence and Link to Merlin/NF2 Signaling
Source: Front Oncol. 2020 May 7;10:687. doi: 10.3389/fonc.2020.00687 (PMC7221161; doi:10.3389/fonc.2020.00687)

Supplementary figures for:

TGFβ signaling increases net acid extrusion, proliferation and invasion in Panc-1 pancreatic cancer cells: SMAD4 dependence and link to Merlin/NF2 signaling

Malinda, R.R., Zeeberg, K., Sharki, P.C., Ludwig, M.Q,

Pedersen, L.B., Christensen, S.T., and Pedersen, S.F.[#]

Section for Cell Biology and Physiology, Department of Biology, Faculty of Science, University of Copenhagen, Universitetsparken 13, DK-2100 Copenhagen, Denmark

Legends to supplementary figures

Suppl. Figure 1. Knockdown of NHE1 or NBCn1 does not prevent EMT induction in Panc-1 cells

A. Panc-1 cells were treated with siRNA against NHE1 or NBCn1 as indicated, treated or not with TGFβ as above, and blotted for E-cadherin, CTGF, NHE1 and NBCn1, with GAPDH and DCTN1 as loading controls as shown. B (E-cadherin) and C (CTGF) show the corresponding quantified data (mean with S.E.M. error bars), normalized to the level in mock-transfected cells under control conditions. Data represent 4 independent experiments per condition.

Suppl. Figure 2. pRb and p53 expression in PDAC cells in absence and presence of TGFβ treatment

IFM analysis illustrating the localization and expression level of p-pRb (green) and p53 (red) in Panc-1 (left) and BxPC-3 (right) cells. Nuclei are stained using DAPI. Representative of at 2-3 independent experiments per condition and protein.

Suppl. Figure 1

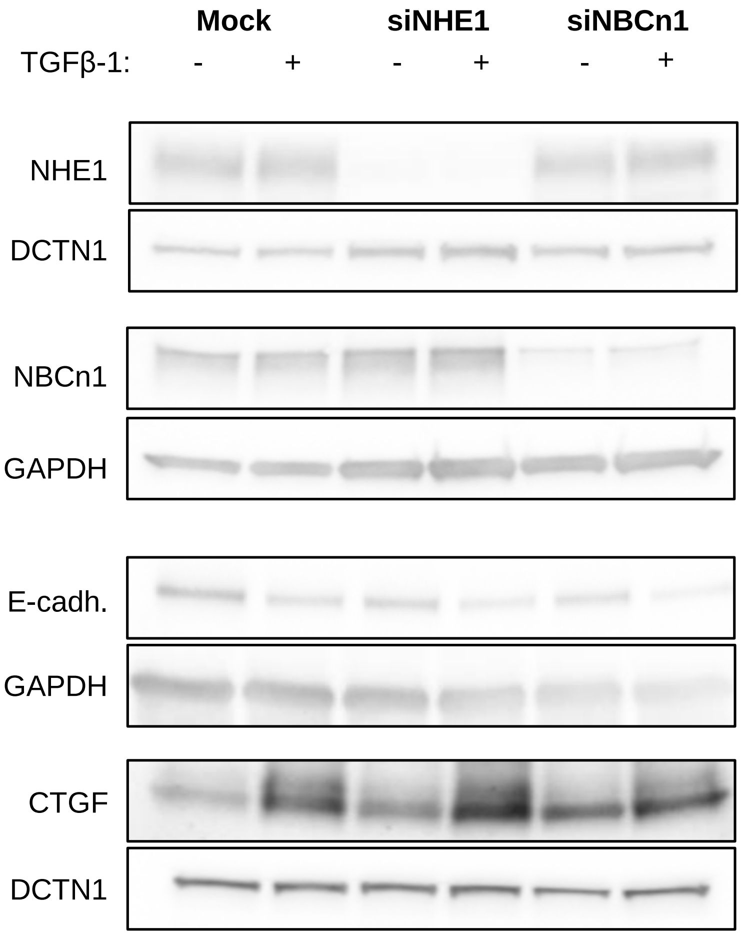

Suppl. Figure 2

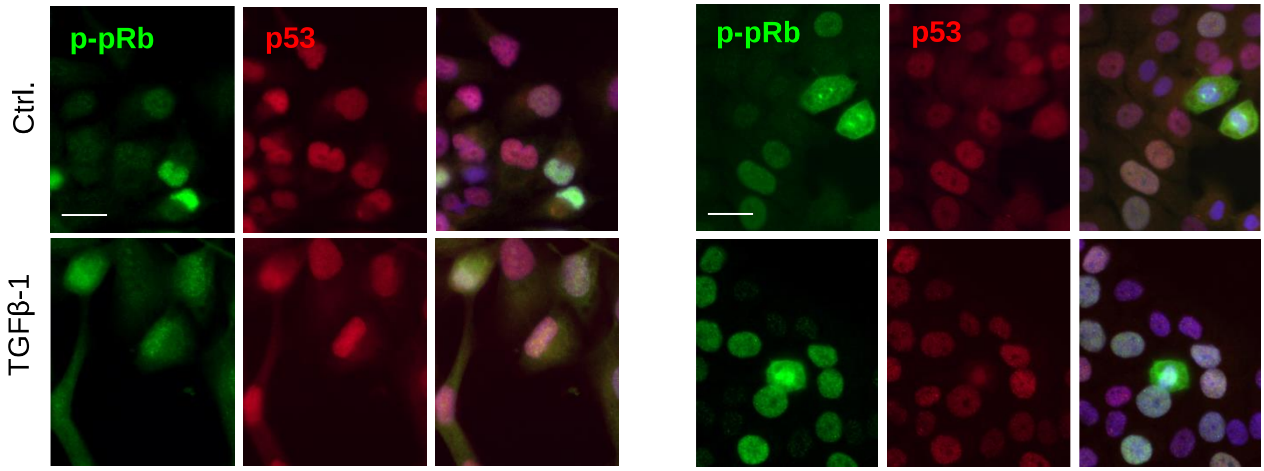

Supplement: Supplementary file 1 [file Data_Sheet_1.PDF]
